# Knowledge, experiences, and perceptions relating to obesity management among primary care physicians in the Lazio Region, Italy

**DOI:** 10.3389/fendo.2023.1249233

**Published:** 2023-11-10

**Authors:** Valeria Guglielmi, Danila Capoccia, Benedetta Russo, Carla Lubrano, Stefania Mariani, Eleonora Poggiogalle, Giuseppe Furia, Aurora Heidar Alizadeh, Cristina Patrizi, Martina Sapienza, Gianfranco Damiani, Maria Grazia Tarsitano, Caterina Conte, Simona Frontoni

**Affiliations:** ^1^ Department of Systems Medicine, University of Rome Tor Vergata, Rome, Italy; ^2^ Internal Medicine Unit - Obesity Center, University Hospital Policlinico Tor Vergata, Rome, Italy; ^3^ Italian Obesity Society (SIO), Pisa, Italy; ^4^ Department of Medical Surgical Sciences and Biotechnologies, Sapienza University of Rome, Santa Maria Goretti Hospital, Latina, Italy; ^5^ Unit of Endocrinology, Diabetes and Metabolism, Fatebenefratelli Gemelli Isola Hospital, Rome, Italy; ^6^ Department of Experimental Medicine, Section of Medical Pathophysiology, Food Science and Endocrinology, Policlinico Umberto I, Sapienza University of Rome, Rome, Italy; ^7^ Directive Council of Order of Physicians and Dentists of the Province of Rome, Rome, Italy; ^8^ Local Health Authority Roma 1, Hospital Management Area, Rome, Italy; ^9^ Department of Life Sciences and Public Health, Università Cattolica del Sacro Cuore, Rome, Italy; ^10^ Department of Woman and Child Health and Public Health, Fondazione Policlinico Universitario “A. Gemelli” Istituto di Ricovero e Cura a Carattere Scientifico (IRCCS), Rome, Italy; ^11^ Department of Medical and Surgical Science, University Magna Grecia, Catanzaro, Italy; ^12^ Department of Human Sciences and Promotion of the Quality of Life, San Raffaele Roma Open University, Rome, Italy; ^13^ Department of Endocrinology, Nutrition and Metabolic Diseases, Istituto di Ricovero e Cura a Carattere Scientifico (IRCCS) MultiMedica, Sesto San Giovanni, Italy

**Keywords:** obesity, primary care providers, survey study, experiences, management

## Abstract

**Background:**

Primary care providers (PCPs) play an essential role in obesity care as they represent the first contact for patients seeking weight loss interventions.

**Objective:**

This study explored the knowledge, experiences, and perceptions of PCPs in the Lazio Region of Italy in the management of obesity.

**Design and subjects:**

We conducted an anonymous survey delivered from March to July 2022 via the newsletter of Rome Provincial Order of Physicians and Dentists and at the annual meeting of the regional section of the Italian Obesity Society.

**Approach:**

The survey consisted of 24 closed-ended questions grouped into 5 sections: sociodemographic and work information; assessment of obesity; management of obesity; connections with regional Centres for Obesity Management; attitudes towards obesity.

**Key results:**

A total of 92 PCPs accessed the survey. Of those, 2.2% were excluded because they did not see any patients with obesity. A total of 68 PCPs (75.6%) had complete questionnaires and were included in this analysis. All participants reported asking their patients about their eating habits, lifestyle, and clinical complications at the first assessment. Body weight and blood pressure were measured by 98.5% of participants and 82% calculate body mass index (BMI), while a small proportion of PCPs analysed body composition and fat distribution. Over 80% prescribed laboratory tests and ECG. Approximately 40% of PCPs did not refer patients for nutritional counselling, and most prescribed a low-calorie diet. Sixty-three percent referred patients to an endocrinologist, 48.5% to a psychotherapist, and a minority to specialists for obesity complications. Twenty-three percent prescribed anti-obesity medications and 46.5% referred patients for bariatric surgery only in severe cases. Ninety-one percent stated that obesity is “a complex and multifactorial disease” and 7.4% considered obesity to be secondary to other conditions.

**Conclusions:**

Despite most PCPs adopt a correct approach to manage patients with obesity, many aspects could be improved to ensure optimal and multidisciplinary management.

## 1 Introduction

Obesity is a chronic, relapsing and multifactorial disorder associated with reduced quality of life and characterized by an abnormal or excessive body fat accumulation leading to a significantly increased risk for several chronic diseases, such as diabetes, cardiovascular diseases (CVDs), depression and cancer that contribute to increased health care costs and a significant reduction in life expectancy (1, 2).

The prevalence of overweight and obesity in adults has reached epidemic proportions worldwide. The World Health Organisation (WHO) estimates that 59% of adults are living with overweight or obesity, and almost a quarter (23%) of adults in the European Region are living with obesity. The highest prevalence of both overweight and obesity are found in Mediterranean and Eastern European countries. Italy has less alarming levels of obesity and overweight than other European countries among the adult population, the prevalence of excess weight being southern regions higher (3). According to the most recent ISTAT (National Statistical Institute) estimates, in 2018 in Italy one in four minors are living with overweight or obesity and the share almost doubles among adults (46.2% among people aged 18 and over) (4). Sustained weight loss in patients with obesity is associated with the prevention, alleviation, and resolution of obesity-related comorbidities (5).

Primary care providers (PCPs) play an essential role in obesity care and should work to promptly identify cases, initiate treatment, and forward to specialist services where appropriate. They are often the primary contact for patients seeking either medical or surgical weight loss interventions. Patients who see their PCP at least once per year are more likely to undergo evidence-based preventative interventions such as vaccination, colonoscopy or mammography (6). International guidelines suggest that PCPs opportunistically screen and help patients engage in weight loss programs (7, 8). A recent study demonstrated that a practical primary care-based method to provide guidance on a low-carbohydrate diet led to a mean weight loss of 10 kg and improved diabetic control in 97% of those interested in the approach, with sustained results for nearly 3 years (9).

This study explores the knowledge, experiences, perceptions and educational needs of PCPs in the Lazio Region, central Italy, in the management of patients with obesity by the use of a multiple-choice questionnaire.

## 2 Methods

### 2.1 Study design and setting

We conducted an anonymous survey study from 15 March to 15 July 2022 to investigate the obesity-related knowledge, experiences, perceptions and educational needs of PCPs practicing in the Lazio Region.

The online version of the survey was created using the “LimeSurvey^®^” platform (LimeSurvey Gmbh). The link was sent via a newsletter to physicians practicing in the Province of Rome by the Rome Provincial Order of Physicians and Dentists (OMCEO Rome), and delivered to physicians of other provinces of the Lazio Region by the regional section of the Italian Obesity Society (SIO Lazio) during the promotion of its annual meeting.

The study was conducted according to the requirements of the Declaration of Helsinki and the data collected were processed according to EU Regulation No. 2016/679 (GDPR), Legislative Decree No. 196/2003 “Code on the Protection of Personal Data” and the subsequent amendments, and all the current legislation on data processing and protection. The start page of the online survey detailed the purpose of the survey, and informed on the anonymous nature of the survey. Potential participants could then leave the page or continue to the survey. No information that could render the responders identifiable was collected. Ethical approval was determined to be non-essential for a study of anonymous nature not involving patients, based on regulatory standards and precedent (10–12).

### 2.2 Questionnaire

The survey consisted of 24 closed-ended questions based on a literature review and consensus among the researchers. The questions were grouped into five sections: (1) sociodemographic characteristics and work information (age category, gender, medical specialty); (2) assessment of the patient with obesity; (3) management of obesity; (4) knowledge and connections with regional Centres for Obesity Management; (5) attitudes towards obesity, tested using both questions specifically developed for the questionnaire and questions derived from tests assessing explicit attitudes or stereotypes (13). No information that could render the subject identifiable was collected.

Only the participants who completed the questionnaire were included in the analyses.

### 2.3 Statistical analysis

Descriptive statistics were obtained for all study variables. Categorical variables were summarised as counts and percentages. Statistical analysis was conducted using IBM SPSS Statistics (IBM SPSS Statistics for Windows, Version 28.0. Armonk, NY: IBM Corp.).

## 3 Results

### 3.1 Sociodemographic characteristics and work information

**Figure 1:**
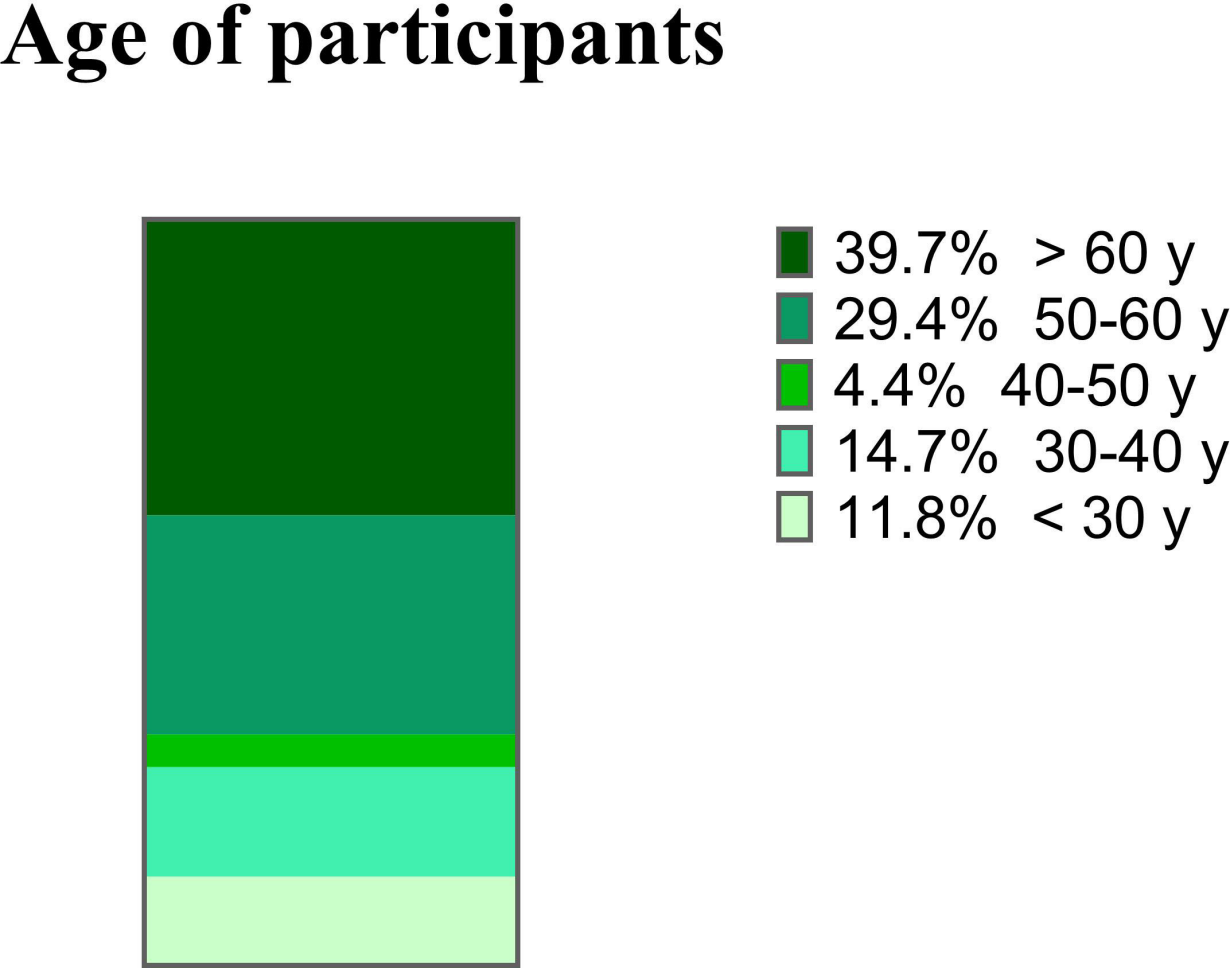
Participant distribution according to age category.

A total of 92 PCPs accessed the survey. Of these, two (2.2%) reported not seeing patients with obesity in their clinical practice, and were excluded from the analyses. Complete questionnaires were available for 68 (75.6%) participants who regularly saw patients with obesity, and were included in this analysis. Most participants (60.3%) were female and over 50 years of age (**Figure 1**).

Most PCPs (77.9%) specialised in primary care only, and the remainder had other specialties, including general surgery (4.4%), endocrinology, obstetrics and gynaecology, nutrition (2.9% each), emergency medicine, haematology, pharmacology, aerospace medicine, forensic medicine, and clinical neurophysiology (1.5% each). The majority (54.4%) had panels of ≥1,000 or more patients. Nearly half (48.6%) of the participants reported having between 50 and 200 patients with obesity (BMI ≥30 kg/m^2^) on their panel, 45.6% had fewer than 50 patients with obesity, and a minority (5.9%) reported having more than 200 patients with obesity on their panel.

### 3.2 Assessment of the patient with obesity

**Figure 2:**
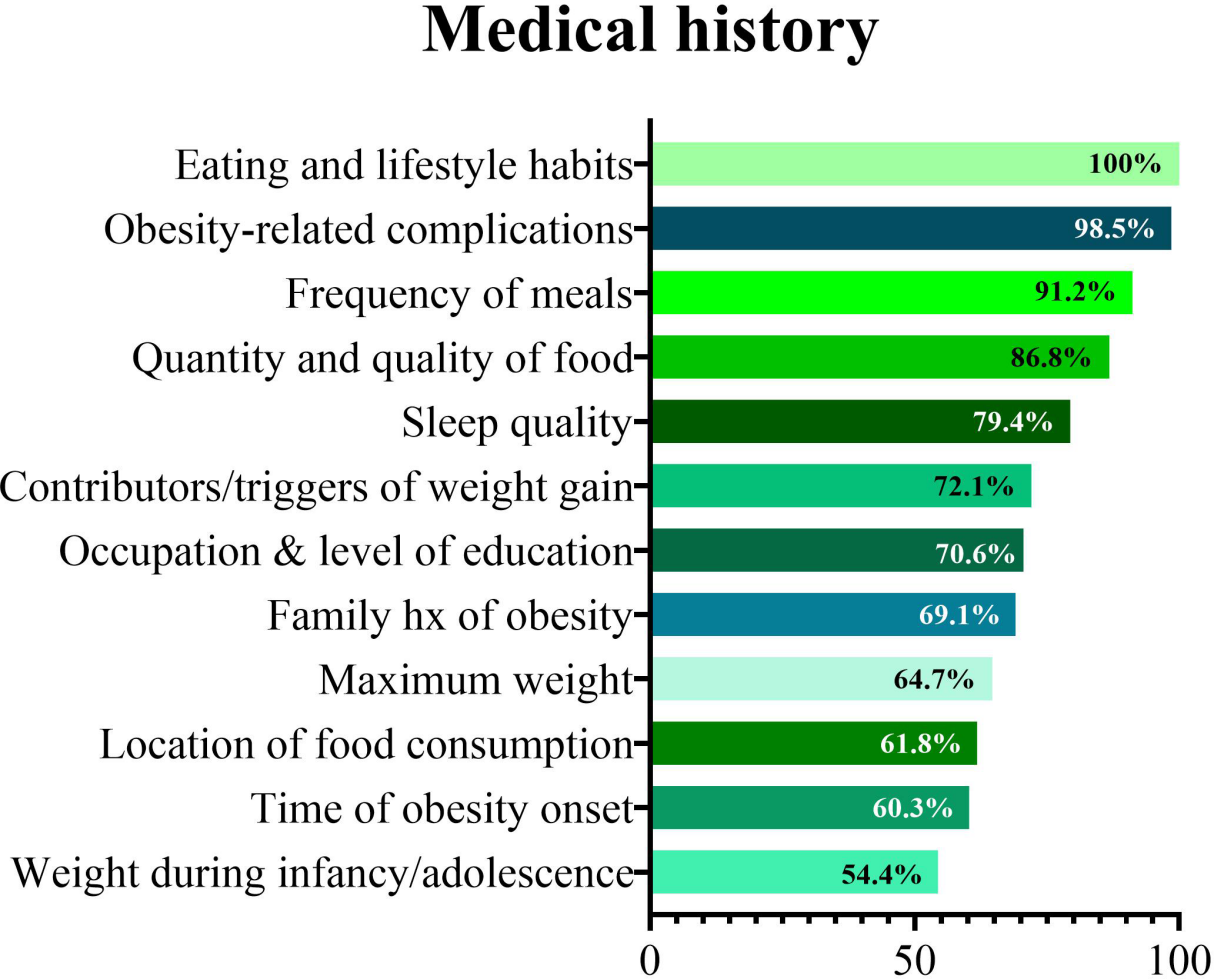
Information collected during by primary care physicians first assessment of a patient with obesity.

Participants were questioned about the information collected during the first assessment of a patient with obesity (**Figure 2**). All participants reported asking patients with obesity about their eating habits and lifestyle. The majority of PCPs investigated the frequency of meals, the dietary quality and quantity of the food consumed and the quality of sleep (**Figure 2**). Nearly all collected information about obesity-related complications. A small, but relevant proportion of PCPs reported asking patients with obesity about other relevant aspects related to the disease. Weight history during childhood and adolescence was the least often collected information.

**Figure 3:**
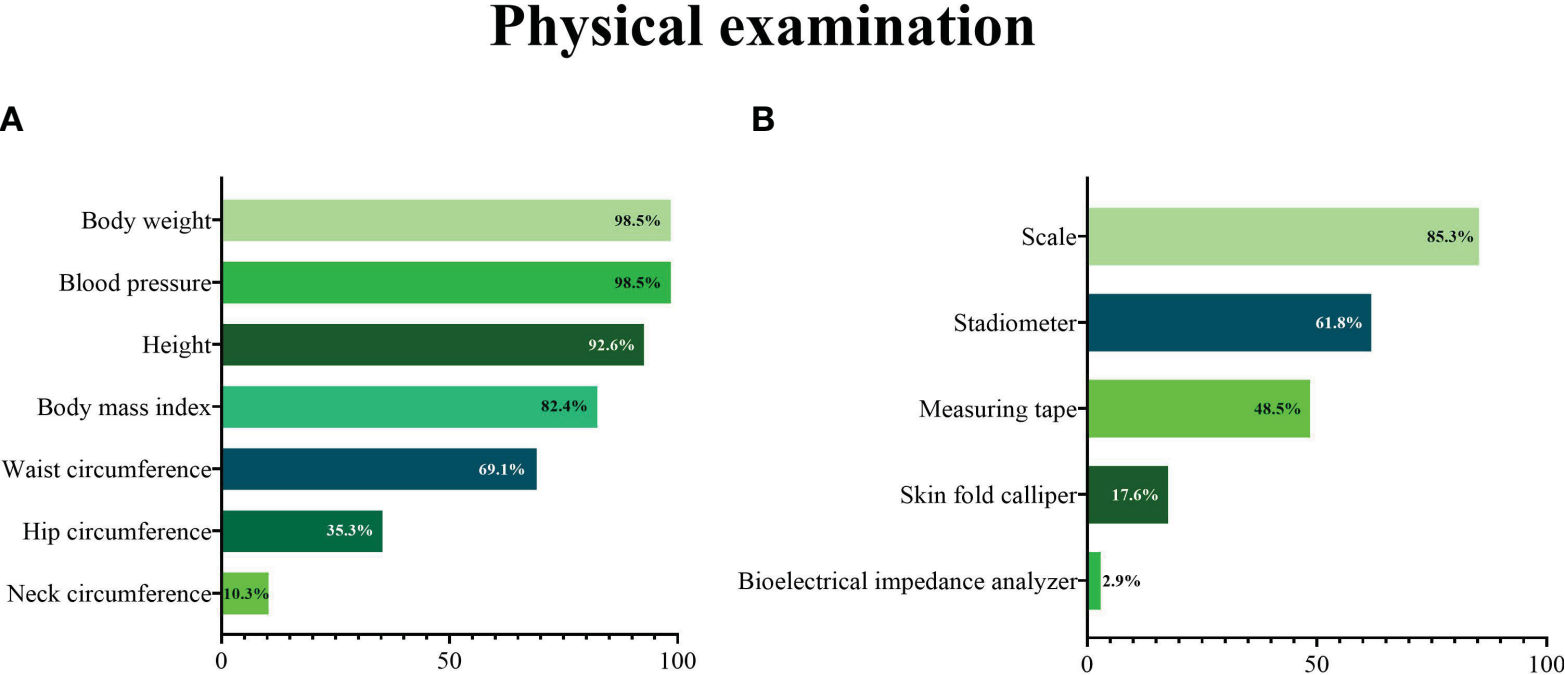
Measurements taken **(A)** and tools used **(B)** during the physical examination by primary care physicians at a first assessment of a patient with obesity.

A further set of questions was related to physical examination during the first assessment of a patient with obesity (**Figure 3**). Nearly all participants reported measuring body weight and blood pressure. Height was measured by 93% of participants, but BMI was computed only by 82% of respondents (**Figure 3A**).

Participants were then asked whether they assessed fat distribution (waist-to-hip ratio) and body composition of patients with obesity. The majority (61.7%) reported measuring only body weight or computing BMI, 32.4% reported measuring waist circumference or both waist and hip circumferences, without assessing body composition, 4.4% reported assessing both body composition and fat distribution, and only one participant reported measuring body composition but not fat distribution.

Most participants reported using a scale during a first assessment of a patient with obesity (**Figure 3B**), although this proportion was lower than those reporting measuring body weight. Similarly, a stadiometer and a measuring tape were used by a lower proportion of participants than those who reported measuring height or waist circumference. A minority of participants reported using instruments for the assessment of body composition (skin fold caliper or bioelectrical impedance analyser).

**Figure 4:**
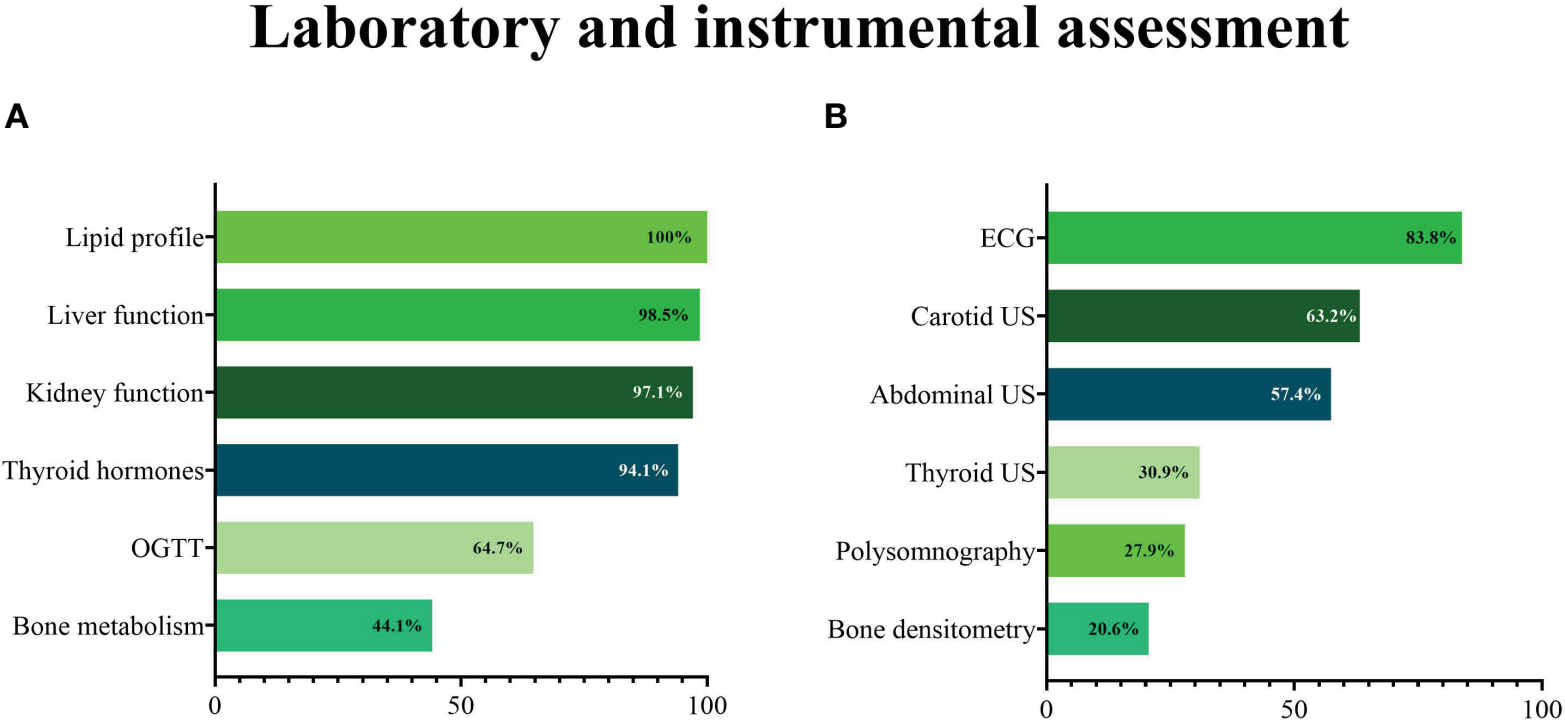
Blood **(A)** and other diagnostic **(B)** tests prescribed by primary care physicians during a first assessment of a patient with obesity. ECG, electrocardiogram; OGTT, oral glucose tolerance test; US, ultrasound.

All PCPs reported prescribing blood testing to assess the lipid profile, and nearly all reported evaluating liver and kidney function (**Figure 4A**). An ECG was the most commonly prescribed diagnostic test, followed by carotid ultrasound and abdominal ultrasound (**Figure 4B**). **Figure 4** depicts the proportions of PCPs prescribing obesity-related blood tests and other diagnostic tests.

### 3.3 Management of obesity

PCPs were also asked about obesity management. Most respondents reported referring patients with obesity to a physician nutrition specialist (27.9%), dietician (25.0%) or endocrinologist (10.3%) for dietary recommendations and prescription of a diet plan. The remainder reported not referring the patient to other healthcare professionals for nutrition counselling (20.6% reported providing a tailored plan, 16.2% a pre-defined, non-personalised dietary scheme). Low-calorie (92%), low-glycemic index (72.0%), and low-carbohydrate (56.0%) were the most popular diets among PCPs providing their patients with obesity with a dietary plan, whereas only a minority reported prescribing very low-calorie diets (8.0%) or very low-calorie ketogenic diets (12.0%).

Approximately one third (36.8%) of the PCPs reported using nutritional supplements or pharmacotherapy for obesity. Specifically, use of anti-obesity medications (AOM) was reported by 23.5%. Orlistat was the most prescribed AOM (17.6%), followed by liraglutide 3 mg (5.9%) and naltrexone/bupropion (2.9%).

**Figure 5:**
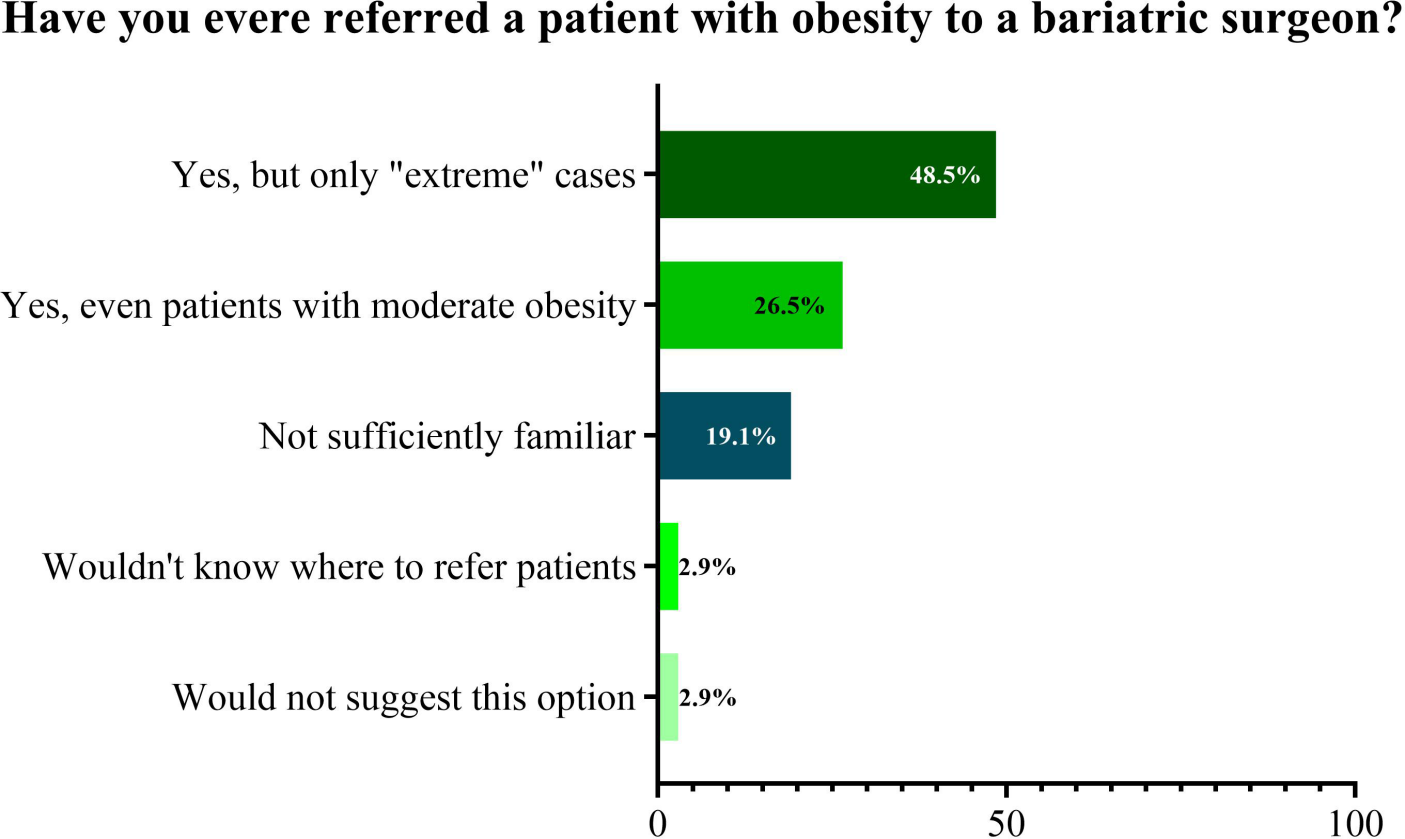
Referral to bariatric surgery by primary care physicians.

PCPs were asked about specialty referrals. Patients with obesity were most referred to an endocrinologist (63.2%), psychotherapist (48.5%), or cardiologist (35.3%). Only a minority of PCPs reported referring their patients with obesity to a pneumonologist (8.8%), gastroenterologist (7.4%), orthopedic surgeon (5.9%), or gynecologist (2.9%). When asked about referrals to bariatric surgery centers, relatively few PCPs were confident regarding the surgical management of obesity (**Figure 5**). The participants’ knowledge of Centers for Obesity Management accredited by the Italian Obesity Society was also investigated.

### 3.4 Knowledge and connections with regional Centres for Obesity Management

Less than half of participants (45.6%) were aware of such Centers. Of these, only 22.6% regularly referred patients to one of the accredited Centers for Obesity Management, 41.9% reported that patient access to care was very difficult and 35.5% stated that they had never inquired about how to refer patients despite being aware of the Centers. The remainder of participants (54.4%) had never heard about this option.

### 3.5 Attitudes towards obesity

**Figure 6:**
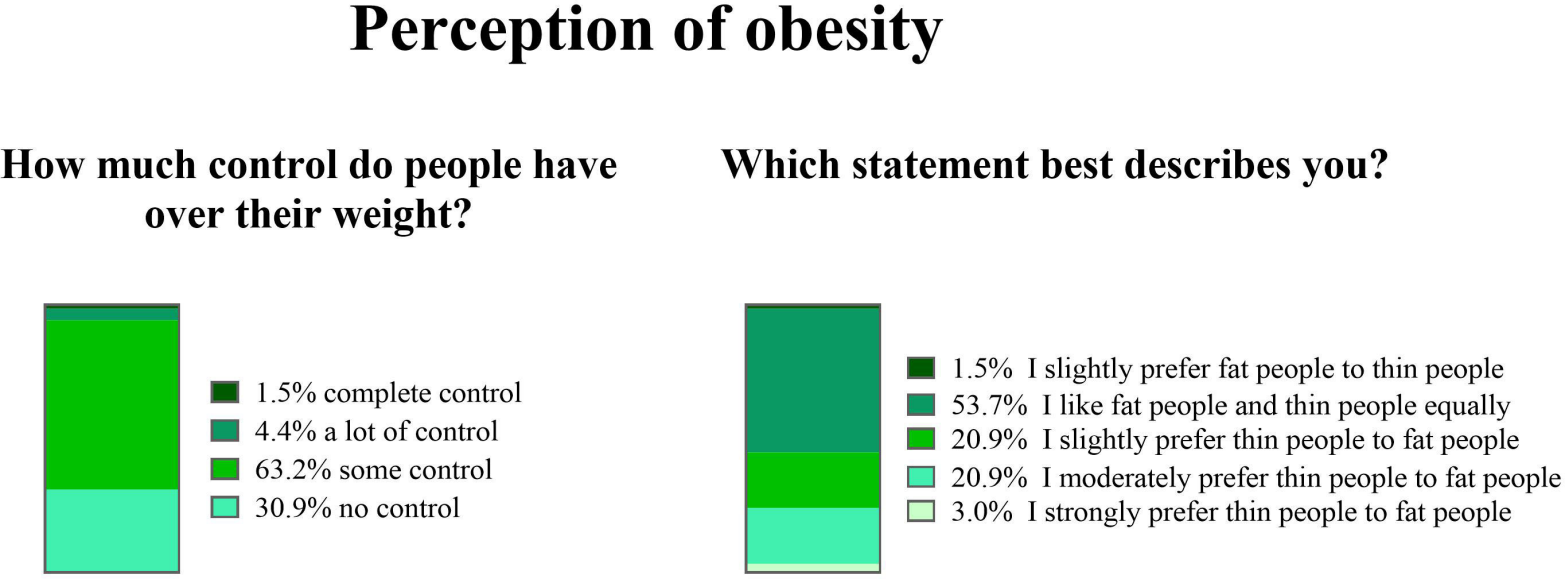
Perception of obesity by primary care physicians.

Most (91.2%) participants stated that obesity is “a complex and multifactorial disease”. However, 7.4% of them deemed obesity as “often secondary (to hormonal dysfunction, iatrogenic, to eating disorders…)”, and one (1.5%) stated that “obesity is not a real disease”. When asked “how much control do people have over their weight?” most participants replied “some control”, and only a minority indicated that people have complete control over their weight (**Figure 6**). More than half of participants expressed no preference for people with normal weight or obesity, but nearly all the remainder had some preference for lean people (**Figure 6**). A multidisciplinary approach was indicated by 75% of participants as the most effective strategy to achieve and maintain weight loss, whereas 25% chose “diet and physical activity” as the best approach.

## 4 Discussion

In this study, we explored the knowledge, experiences, perceptions, and educational needs of PCPs from the Lazio Region, central Italy, in managing patients with obesity.

### 4.1 Assessment of the patient with obesity

At the first assessment of a patient with obesity, all participants reported asking patients about their eating habits, lifestyle and obesity clinical complications. Most investigated the details of meals, food choices and sleep quality. Although most of the participants asked about the presence of a family history of obesity, few inquired about the time of obesity onset and the body weight history in childhood and adolescence. Therefore, even though bad eating habits, sleep disturbances and family history of obesity are identified as major predictors of obesity, the negative impact of obesity duration on the risk of developing obesity cardio-metabolic complications still lacks adequate recognition. Over the years, studies have suggested that living with obesity for a long time increases mortality, regardless of current BMI (14), and that delaying the onset of obesity can lower the risk of developing future cardiovascular disease (CVD) (15, 16). Unfortunately, the diagnosis and treatment of obesity in the primary care setting seems to be declining. The results of a national survey in USA found that, relative to 2008–2009, height and weight were more likely to be measured in primary care visits occurring between 2012–2013 (54% *vs*. 73% of visits, respectively). However, approximately 5% fewer patients with a BMI of 30 kg/m^2^ or higher received a diagnosis of obesity. Only 21% of patients with obesity were provided with weight-related counselling in 2012–2013, compared with 33% in 2008–2009 (17).

In contrast with the high proportions of participants who reported to measure patient body weight (98.5%) and height (93%), only 85.3% of them actually had a scale and 61.3% a stadiometer, prompting that approximately 15% of PCPs rely on self-reported weight. In a previous research about primary care practice, on 707,819 electronic medical records (EMRs) of Canadian adult patients aged 40 and older, only 48.7% had 1 or more BMI values recorded and 11.5% had at least 1 waist measurement recorded (18). Research comparing self-reported with measured weight and height data has generally found discrepancies, with individuals having overweight/obesity more frequently under-reporting weight with a consequent underestimation of BMI and misclassification of individuals (19, 20).

BMI was computed by 82% of respondents, but fewer measured indicators of body fat distribution such as waist circumference. Failure to include waist circumference in clinical routine assessments prevents taking advantage of a simple tool that provides independent and additive information to BMI for predicting risk of type 2 diabetes mellitus, cardiovascular events and mortality (21). Neck circumference was the anthropometric collected least often, despite its utility in stratifying obstructive sleep apnoea (OSA) risk (22–24). Few PCPs reported using instruments for body composition analysis such as a bioelectrical impedance analyser, which can evaluate nutritional status and provide an estimate of the patient’s fat and fat-free mass. Body composition analysis allows more accurate monitoring of the weight loss during dietary interventions, especially for those PCPs (36.8%) personally providing the patients with a dietary plan. Indeed, weight tracking and BMI do not provide insight into the relative contributions of fat and lean mass and their changes to the obesity-related risks.

Most respondents reported prescribing laboratory tests to assess lipid profile, liver and renal function, and glucose metabolism. The high awareness of the risk of developing obesity-associated cardio-metabolic complications is also confirmed by the high prescription of cardiovascular diagnostic tests (ECG and carotid ultrasound) and liver ultrasound. Although the relatively low rate of cardiology referrals (35%) may seem inconsistent with these data, it is possible that this reflects judicious referral, i.e. limited to patients with abnormal cardiovascular diagnostic tests. At the first visit of a patient with obesity, the majority of PCPs reported testing thyroid hormones, and almost one-third prescribed a thyroid ultrasound. Although at the first assessment of a patient with obesity thyroid function test (thyroid-stimulating hormone level) is recommended (25), a further endocrine evaluation is indicated only when there is clinical suspicion of thyroid disease. Obesity is a chronic metabolic and multifactorial disease that involves complex interactions between genetic, biological, behavioural, social and environmental factors, and that only rarely can be ascribed to secondary causes like endocrine disorders. In this view, the prescription of thyroid ultrasound may reflect a partial acknowledgement of obesity as a primary disease (26, 27).

The fact that almost half of the participants investigated bone metabolism but only 20% prescribed a bone densitometry testifies the PCPs awareness of the negative impact of obesity on bone health and the recognition of the role of altered bone quality parameters (circulating bone turnover markers and bone microarchitecture and strength by advanced imaging techniques), rather than bone mineral density (BMD assessed by DXA), as major determinants of bone fragility in obesity (28). This is in line with a previous survey in which, among 107 respondents, less than 10% used evidence-based guidelines to inform obesity treatment decisions (29).

### 4.2 Management of obesity

Participants were asked questions regarding dietary advice, medication use, request for specialists advice and use of bariatric surgery.

Just over 60% of the participants reported that they regularly refer patients with obesity to a physician nutrition specialist or dietitian or endocrinologist, while a good proportion answered that they self-manage patients by using diet plans prepared by themselves. This is in contrast with what is reported by the guidelines for the treatment of the patient with obesity, which requires a multidisciplinary approach (30).

The most commonly used diets are low-calorie diets or low-carbohydrate diets. Only a minority reported using very low calorie ketogenic diets (VLCKD). There is probably a lack of knowledge on the part of PCPs about some diet programs, such as the VLCKD, which has been recently proposed as an appealing nutritional strategy for management of obesity and its associated complications (31). Almost 50% of PCPs plan to refer patients with obesity to a psychotherapist, probably recognizing the close association between obesity and psychological disorders. This association is not only linked to physical health outcomes, however, as obesity has been extensively associated with mental illness. Both obesity and severe mental illness decrease quality of life and are associated with an increase in disability, morbidity, and mortality, and when they occur together, these adverse health outcomes are magnified. Despite educational campaigns, increased awareness, and improved treatment options, the high prevalence of mental illness and comorbid obesity remains a serious problem (32). Few studies in the literature have highlighted the improvement in obesity treatment when PCPs also use therapeutic strategies to treat psychological aspects. Sarto et al. showed the effectiveness of a mindfulness eating programme to reduce emotional eating in adults with overweight/obesity in primary care settings and change the relationship with food in patients suffering from overweight or obesity (33).

Several AOMs have been developed over the last decades, albeit with limited success, until recently. Currently available agents include centrally acting appetite suppressants and peripherally acting compounds. In Italy, three AOMs are available: orlistat, liraglutide 3 mg and naltrexone/bupropion. About 25% of PCPs reported prescribing drug therapies, mostly orlistat. According to Italian prescribing rules, PCPs can prescribe orlistat and liraglutide 3 mg. PCPs have been authorized to prescribe the latter only a couple of years ago, whereas naltrexone/bupropion can only be prescribed by specialists managing patients with obesity (endocrinologists, cardiologists, internal medicine physicians, nutrition specialists). These regulatory criteria likely contribute to PCPs’ reluctance to prescribe AOMs, but therapeutic inertia may also play a role (34). As regards the referrals to bariatric surgery, nearly half of PCPs refer patients for bariatric surgery only in cases of severe obesity and a third in cases of moderate obesity. Over 20% do not recommend surgery, mostly due to lack of knowledge. Most PCPs are aware of weight loss information sessions and bariatric services provided within our integrated health network, but almost one third were unable to identify a bariatric surgeon. This is consistent with data from Italy showing that people with obesity self-refer to bariatric centres and rarely are referred from PCPs (35, 36), suggesting low compliance with current guidelines on surgery for obesity management. Modern bariatric procedures are supported by strong evidence of efficacy and safety. People with severe obesity - and especially those with type 2 diabetes - should be involved in a shared decision-making conversation about the risks and benefits of bariatric surgery compared with continuing with usual medical and lifestyle treatment (37). Our data confirm the difficulty for PCPs to refer for bariatric surgery, as also reported by other authors (38). On the other hand, there are studies showing that PCPs welcome supportive tools to improve the care of long-term follow-up of bariatric patients and would actively participate in the development of lifelong disease management plans to address the growing number of bariatric patients (39).

### 4.3 Attitudes towards obesity

PCP’s perception of obesity was assessed testing explicit attitudes (13), i.e. preferences, beliefs, and attitudes that people consciously acknowledge, personally endorse, and are able to name and articulate (40). It is worrisome that, although most participants replied “some control” when asked “how much control do people have over their weight?”, some indicated that people have complete control over their body weight. The widespread belief that one can control their body weight and that those who cannot are weak, gluttons, or lazy is the foundation of weight stigma (41), and is in fact associated with explicit weight bias (42, 43). Weight stigma is deeply rooted in the society (44), and the healthcare setting is no exception (45). Forty-five percent of participants in our survey reported having some preference for lean people, suggesting they feel that expressing negative attitudes towards people with obesity is socially acceptable. Avoiding stigmatization should be a pillar of obesity management in primary care, as weight stigma may have detrimental consequences on people living with obesity (7). These include increased risk of depression, further weight gain, avoidance of medical consultation, and even suicide (7). Seven percent of participants in the survey deemed obesity as often secondary to other conditions, and one even stated that “obesity is not a real disease”. Obesity is now recognized as a chronic, relapsing, and progressive disease (46). Nonetheless, 25% of participants indicated “diet and physical activity” as the most effective strategy to achieve and maintain weight loss, which may reflect an over-simplified approach of “eat less, move more” that might be perceived as discouraging by patients with obesity (47), does not address the complexity of the disease, and is therefore discouraged by current guidelines (48, 49). Increasing recognition of obesity as a disease is an effective strategy to reduce weight bias in healthcare professionals (50). Nevertheless, despite the impressive burden of obesity, coverage in medical education is strikingly poor (51, 52), and research on interventions to reduce weight bias is relatively limited. Of note, despite some interventions are successful in mitigating weight bias, their durability is often short (53).

### 4.4 Study limitations and strengths

A limitation of our study is that we only assessed explicit attitudes. Although there is some correlation between explicit and implicit (unconscious) associations (54), the latter may independently predict relevant outcome variables better than parallel self-report measures (13). Our survey aimed to provide a general picture of the obesity-related knowledge, experiences, perceptions and educational needs of PCPs, and was not specifically focused on weight bias. Even so, our data indicate that a relevant proportion of PCPs perceive patients with obesity as responsible for their condition and reported having a preference for normal weight individuals. Gaining awareness of weight bias could be a first step to counteract it. In this light, the Project Implicit, which aims to educate the public about bias towards different topics including weight (https://implicit.harvard.edu/), might help reducing weight bias among PCPs. Further potential limitations include the self-selection bias and the relative small sample size. Furthermore, information on the Province of origin was not collected, and therefore it cannot be confirmed if all areas of the Lazio Region were equally represented. However, this should be considered as a pilot study to provide a first picture of the knowledge, experiences, perceptions and educational needs relating to the management of obesity among PCPs in the Lazio Region. Our results lay the foundations for further initiatives aimed at increasing the acknowledgement of obesity as a disease and improving of obesity care in the Lazio Region. It should also be acknowledged that, besides the impact on patient health and quality of life, obesity poses a huge economic burden on the society. A study that included 161 countries estimated that the economic cost of overweight and obesity is, on average, 2.19% of gross domestic product (GDP). This cost is considerable in diverse geographic and economic circumstances (55). The authors also estimated that, if current trends continue, the economic impact will climb to approximately 3.29% of projected GDP on average in 2060, with the greatest increase in limited-resource countries. In Italy, the total costs attributable to obesity amounted to €13.34 billion in 2020 (56). A more effective management of obesity starting from primary care could have a major economic impact. Although Primary Care is theoretically the optimal place for providing weight management counselling, consultation length of PCPs is often short, lasting only few minutes (57). Preventative counselling is time-consuming. A PCP with a 2,500-patient panel would require 4.1 hours per day to provide obesity counselling, dietary and obesity counselling being the most time-consuming tasks (58). Advice on physical activity, which our survey only partially addressed, is another crucial element in managing obesity (7, 59). However, only a small number of PCPs offer this advice due to time constraints, insufficient adherence, competing priorities, and insufficient knowledge (60). This highlights the need to enhance referral to specialists in obesity care and to establish a group of healthcare practitioners dedicated to obesity care. A nutritionist or dietician, an expert in physical activity, a psychiatrist or psychologist, and a nurse should be part of such group (7). In fact, there is a need to improve care coordination (61). Intercommunication between PCPs and specialists is essential for high-performing primary care, reducing fragmentation, inefficiencies, and healthcare costs (62). The use of a multidisciplinary team in a network system is also recommended by the European Practical and Patient-Centred Guidelines for Adult Obesity Management in Primary Care (7). Of note, the European Association for the Study of Obesity (EASO) has designed a leaflet, tailored to PCPs, to be used as a practical tool for reviewing information on obesity management (63).

A strength of our study is the comprehensive assessment of diverse aspects of obesity management among PCPs, spanning from patient assessment to interaction with Centres for Obesity Management, and attitudes towards obesity. Another relevant aspect is the collaboration of a regional section of SIO and a Provincial Order of Physicians and Dentists. This shared effort lays the foundation for building a network of healthcare professionals involved in obesity care.

### 4.5 Conclusions

In conclusion, our data indicate that, despite most PCPs demonstrate some understanding of the complex nature of obesity and adopt a correct approach to assess patients with obesity on the first visit, many aspects could be improved to ensure that patients with obesity receive multidisciplinary management for their complex disease. Future interventions should aim at improving individual PCP and practice staff education, and implementing a referral network of specialist services and multidisciplinary team Centers for Obesity Management. Ultimately, reducing discrimination will help fully integrate anti-obesity services in our healthcare systems.

## Data availability statement

The raw data supporting the conclusions of this article will be made available by the authors, without undue reservation.

## Ethics statement

Ethical approval was not required for the studies involving humans because the survey was anonymous and conducted according to the requirements of the Declaration of Helsinki and the data collected were processed according to EU Regulation No. 2016/679 (GDPR), Legislative Decree No. 196/2003 “Code on the Protection of Personal Data” and the subsequent amendments, and all the current legislation on data processing and protection. No information that could possibly render responders identifiable was collected. The studies were conducted in accordance with the local legislation and institutional requirements. Written informed consent for participation was not required from the participants or the participants’ legal guardians/next of kin in accordance with the national legislation and institutional requirements because the start page of the online survey detailed the purpose of the survey, and informed on the anonymous nature of the survey. Potential participants could then leave the page or continue to the survey. Ethical approval was determined to be non-essential for a study of anonymous nature not involving patients, based on regulatory standards and precedent [see refs 10; 11; 12 in the manuscript].

## Author contributions

VG, DC, MGT, CC and SF designed the work. GF, AA, CP, MS, GD contributed to data acquisition; VG, DC and CC analysed the data and drafted the work. VG, DC, BR, CL, SM, EP, MGT, CC, SF contributed to data interpretation. CL, SM, EP, GF, AA, CP, MS, GD revised the work critically for important intellectual content and provided approval for publication of the content. All authors agree to be accountable for all aspects of the work in ensuring that questions related to the accuracy or integrity of any part of the work are appropriately investigated and resolved.

## Group members of the Rome OMCeO Group

Antonio Magi, Stefano De Lillo, Guido Coen Tirelli.

## In Memoriam

We dedicate this article to Simona Frontoni, a passionate woman, teacher, and researcher who enthusiastically devoted her entire life to her family, students, and patients with diabetes.
